# The M_1_ muscarinic receptor is present in situ as a ligand-regulated mixture of monomers and oligomeric complexes

**DOI:** 10.1073/pnas.2201103119

**Published:** 2022-06-07

**Authors:** Sara Marsango, Laura Jenkins, John D. Pediani, Sophie J. Bradley, Richard J. Ward, Sarah Hesse, Gabriel Biener, Michael R. Stoneman, Andrew B. Tobin, Valerica Raicu, Graeme Milligan

**Affiliations:** ^a^Centre for Translational Pharmacology, Institute of Molecular, Cell and Systems Biology, College of Medical, Veterinary and Life Sciences, University of Glasgow, Glasgow G12 8QQ, United Kingdom;; ^b^Physics Department, University of Wisconsin-Milwaukee, Milwaukee, WI 53211;; ^c^Department of Biological Sciences, University of Wisconsin-Milwaukee, Milwaukee, WI 53211

**Keywords:** G protein-coupled receptor, fluorescence fluctuation analysis, muscarinic receptor, quaternary organization, fluorescence intensity fluctuation spectrometry

## Abstract

The quaternary organization of rhodopsin-like G protein-coupled receptors in native tissues is unknown. To address this we generated mice in which the M_1_ muscarinic acetylcholine receptor was replaced with a C-terminally monomeric enhanced green fluorescent protein (mEGFP)–linked variant. Fluorescence imaging of brain slices demonstrated appropriate regional distribution, and using both anti-M_1_ and anti–green fluorescent protein antisera the expressed transgene was detected in both cortex and hippocampus only as the full-length polypeptide. M_1_-mEGFP was expressed at levels equal to the M_1_ receptor in wild-type mice and was expressed throughout cell bodies and projections in cultured neurons from these animals. Signaling and behavioral studies demonstrated M_1_-mEGFP was fully active. Application of fluorescence intensity fluctuation spectrometry to regions of interest within M_1_-mEGFP–expressing neurons quantified local levels of expression and showed the receptor was present as a mixture of monomers, dimers, and higher-order oligomeric complexes. Treatment with both an agonist and an antagonist ligand promoted monomerization of the M_1_-mEGFP receptor. The quaternary organization of a class A G protein-coupled receptor in situ was directly quantified in neurons in this study, which answers the much-debated question of the extent and potential ligand-induced regulation of basal quaternary organization of such a receptor in native tissue when present at endogenous expression levels.

## Significance

Although it is appreciated that members of the large family of rhodopsin-like cell surface receptors can form dimeric or larger protein complexes when expressed at high levels in cultured cells, their organizational state within native cells and tissues of the body is largely unknown. We assessed this in neurons of the central nervous system by replacing the M_1_ muscarinic acetylcholine receptor in mice with a form of this receptor with an added fluorescent protein. Receptor function was unaltered by this change, and the biophysical approach we used demonstrated that the receptor exists as a mixture of monomers and dimers or oligomers. Drug treatments that target this receptor promote its monomerization, which may have significance for receptor function.

Measuring and understanding the extent and potential significance of quaternary organization of members of the class A (rhodopsin-like) family of G protein-coupled receptors (GPCRs) have both fascinated and frustrated researchers for many years (1, 2). Over time, a wide range of methods have been applied to address this question, and many different GPCRs have been examined. Outcomes have ranged from assertions that such receptors are monomeric and that results consistent with other conclusions reflect either artifacts of the method of measurement or that studies have been performed at nonphysiological levels of expression of the receptor being studied, to those that have suggested rather stable dimeric or tetrameric complexes (1). Only in the case of rhodopsin, the photon receptor expressed at very high levels (in the range of 24,000–30,000 molecules/µm^2^) in rod outer segments of the eye, have detailed studies been conducted in situ on a class A GPCR. In this example, various studies have shown that rhodopsin is organized as rows of dimers (3, 4). However, to our knowledge, no other GPCR is expressed natively at levels akin to rhodopsin. As such, although a substantial number of studies, generally performed in transfected cell lines or in artificial bilayer systems, have provided evidence that other GPCRs can and do form dimeric and/or higher-order quaternary complexes in a concentration-dependent manner (1, 2), how levels of expression required to observe such complexes relate to expression levels in native cells and tissues has been poorly defined, as is the stability of such complexes and whether they are regulated by ligand binding.

Developments in fluorescence fluctuation analysis (FFA) have facilitated efforts to define the oligomeric status of transmembrane receptor proteins (5, 6). Unlike methods based on resonance energy transfer, only a single fluorophore-linked protein is required to be expressed to use FFA. It is, therefore, more practical to use such methods in native cells and tissues if linked to genome-editing approaches and/or the generation of transgenic “knock-in” animal models in which a receptor of interest is replaced with a fluorophore-tagged, modified form of the receptor. Moreover, the recent introduction of fluorescence intensity fluctuation (FIF) spectrometry (7–10) has overcome issues with other methods based on FFA that result in information being compressed due to averaging of oligomer-size data from interrogated regions of interest (RoIs) in which complex mixtures of oligomers of different sizes may be present (7, 8).

To define whether the class A M_1_ muscarinic acetylcholine receptor is present in hippocampal and cortical neurons as strict monomers or as a range of monomeric, dimeric, and, potentially, oligomeric complexes, we applied FIF spectrometry to images of such neurons isolated from a line of transgenic mice in which we replaced the M_1_ receptor with a form of the receptor that includes C-terminally linked monomeric enhanced green fluorescent protein (mEGFP). We first show that both expression levels and function of the introduced M_1_-mEGFP construct appear equivalent to the native M_1_ receptor in wild-type (WT) mice, using a range of methods and measures ranging from [^3^H]ligand binding and cell signaling assays to locomotion. We then demonstrate in hippocampal and cortical neurons that in the basal state, the M_1_-mEGFP construct is present as a mixture of monomers and dimeric or oligomeric complexes. We also show that the presence of either an agonist or an antagonist ligand promotes monomerization of the receptor. In these studies, we combined analysis of images of a fluorophore-modified receptor in situ with calculation of receptor oligomer complexity. The studies provide a clear and unambiguous answer to a long-standing question that has been the subject of considerable debate (11–13) but that has previously been restricted to studies performed on transfected cell lines. Moreover, these studies are a model for subsequent studies for researchers who plan to explore the topic of dimerization of rhodopsin-family GPCRs.

## Results

**Fig. 1.**
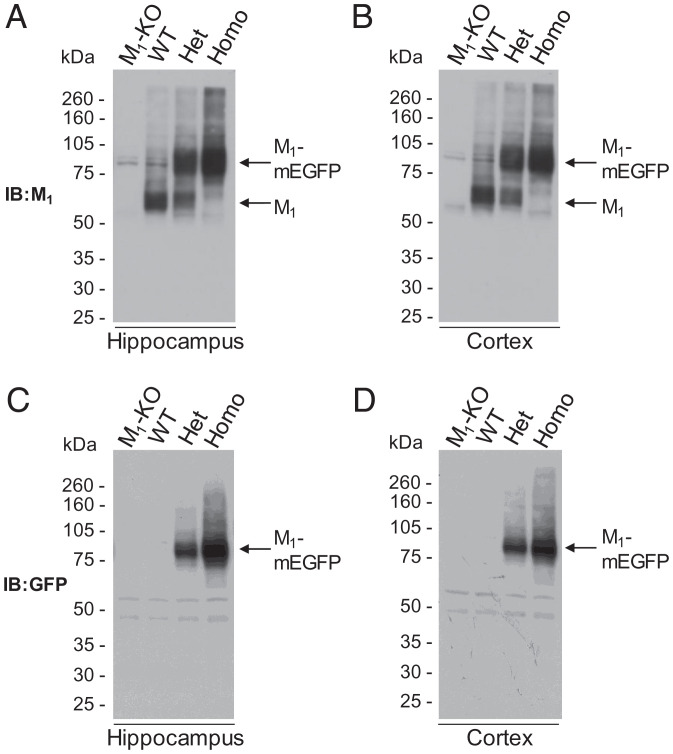
Detection of M_1_-receptor and M_1_-mEGFP in hippocampus and cortex of WT and M_1_-mEGFP transgenic mice. Hippocampus (*A* and *C*) and cortex (*B* and *D*) were isolated from WT, M_1_-knockout (KO) and both homozygous (Homo) and heterozygous (Het) M_1_-mEGFP knock-in mice. Lysates generated from these tissues were resolved by SDS-PAGE and immunoblotted (IB) with antisera able to identify either the M_1_ receptor (*A* and *B*) or mEGFP (*C* and *D*). Representative immunoblots are shown.

To assess directly in native tissue the long-standing debate on the extent of potential quaternary organization of rhodopsin-like, class A GPCRs, we generated a transgenic knock-in line of mice in which the M_1_ muscarinic acetylcholine receptor was replaced with a variant of this receptor in which monomeric (Ala^206^Lys) mEGFP (14) was appended in-frame to the receptor C-terminal tail. This was achieved by insertion of a loxP-stop-loxP cassette containing M_1_-mEGFP into the *Chrm1* endogenous locus. Chimeric mice, generated following insertion of embryonic stem cells into blastocysts that were implanted in pseudopregnant females, were bred with Cre-recombinase–expressing mice to generate animals predicted to express M_1_-mEGFP constitutively in cells and tissues that would normally express the unmodified M_1_ receptor (*SI Appendix*, Fig. 1*A*). Genotyping of such mice identified individuals anticipated to express only WT M_1_, those expected to be homozygous for expression of M_1_-mEGFP, and heterozygotes predicted to express both WT M_1_ and M_1_-mEGFP (*SI Appendix*, Fig. 1*B*). Dissection of cortex and hippocampus from each of these genotypes, followed by preparation of lysates and separation of proteins by sodium dodecylsulfate–polyacrylamide gel electrophoresis (SDS-PAGE), allowed immunoblotting with an anti–M_1_-receptor antiserum (Fig. 1 *A* and *B*). In hippocampus from WT mice, the molecular mass of the detected receptor was approximately 55 kDa, with a degree of microheterogeneity that typically reflects variable *N*-glycosylation and other posttranslational modifications. In tissue from homozygous M_1_-mEGFP animals, the detected construct centered at approximately 80 kDa, again with a degree of microheterogeneity, but with no detectable 55-kDa species, while in heterozygote animals, both the 55-kDa and 80-kDa forms were present. As anticipated, in the heterozygotes, both the 55-kDa WT M_1_-receptor and the 80-kDa M_1_-mEGFP variant were present at observationally lower levels than was the corresponding receptor species in the WT and the homozygous transgenic M_1_-mEGFP knock-in animals (Fig. 1*A*). That these species did, indeed, represent the two forms of the M_1_-receptor was evident, as neither was detected in equivalent hippocampal preparations from M_1_-knockout (M_1_-KO) mice (Fig. 1*A*). Entirely equivalent results were obtained when immunoblotting was performed on tissue isolated from the cortex of such animals (Fig. 1*B*). To further validate these conclusions, following SDS-PAGE, we immunoblotted equivalent preparations with an in-house–generated, anti-GFP antiserum. Now, in both hippocampal and cortical preparations, only the 80-kDa mEGFP-tagged form(s) of the receptor was detected (Fig. 1 *C* and *D*). However, in accord with the results with the anti–M_1_ receptor antiserum, this species was present in higher amounts in tissue from the homozygous than the corresponding heterozygous animals and was not detected in tissue from either WT or M_1_-KO mice (Fig. 1 *C* and *D*). Neither antiserum detected more rapidly migrating fragments that might correspond to cleaved or degraded products of the transgene (Fig. 1 *A*–*D*).

**Fig. 2.**
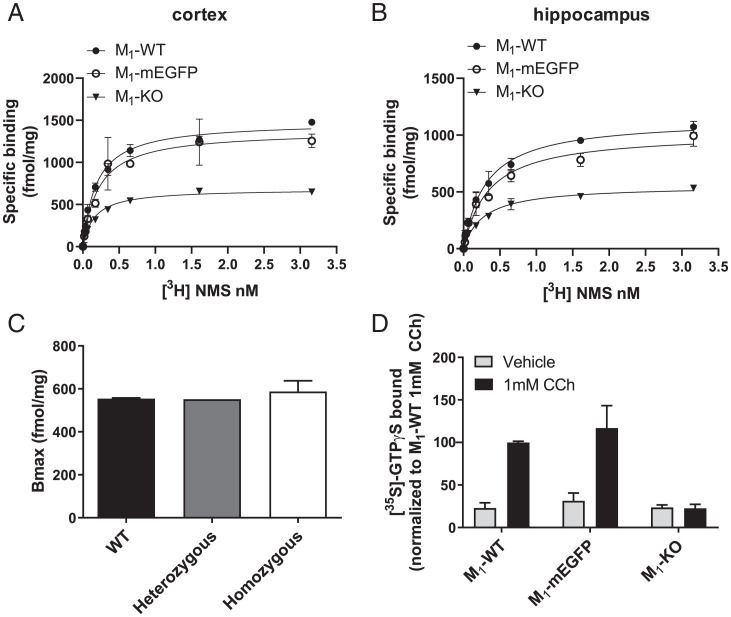
Expression level and function of M_1_-mEGFP in transgenic mice is equivalent to M_1_-receptor in WT animals. (*A* and *B*) Specific binding of various concentration of [^3^H]NMS was assessed in membranes of either cortex (*A*) or hippocampus (*B*) of WT (filled circles, WT), homozygous M1-mEGFP knock-in (open circles, mEGFP), and M_1_-knockout (KO; filled triangles, KO) mice. A representative example of five experiments performed on tissue from different mice is shown with error bars representing the SD of the mean of triplicate data points. (*C*) [^3^H]NMS B_max_ (maximum specific binding) measured in cortical tissue from M_1_-KO mice and subtracted from [^3^H]NMS B_max_ measured in each of WT, heterozygous, and homozygous M_1_-mEGFP knock-in animals. (*D*) Samples equivalent to those used in *A* were employed in [^35^S]GTPγS binding studies performed in the presence of 1 mM carbachol (CCh) (dark bars) or vehicle (light bars). At termination of incubation, samples were subjected to immunoprecipitation with an anti–G_αq_/G_α11_ selective antiserum to focus specifically on contributions of the M_1_ receptor and/or M_1_-mEGFP. Data are presented as mean ± SD (*n* = 3) from separate mice where 100% [^35^S]-GTPγS bound was produced by carbachol in tissue from WT mice.

Immunoblotting provides, at best, semiquantitative observation of relative protein expression levels. To assess directly whether the addition of mEGFP to the C-terminal tail of the M_1_-receptor altered expression level, we performed saturation ligand-binding studies using the muscarinic antagonist [^3^H]*N*-methylscopolamine ([^3^H]NMS) (Fig. 2 *A* and *B*). Measured numbers of specific [^3^H]NMS binding sites were not different in tissue from WT and homozygous M_1_-mEGFP–expressing mice (Table 1). As there are five subtypes of muscarinic receptor (15), all of which bind [^3^H]NMS with similar affinity, to define levels of expression of only the M_1_-receptor, we also measured specific binding of [^3^H]NMS in tissue from M_1_-KO mice (Fig. 2 *A* and *B* and Table 1) and subtracted this from the specific binding in tissue from WT, heterozygous, and homozygous M_1_-mEGFP–expressing mice (Fig. 2*C*). [^3^H]NMS binding corresponding to the M_1_ receptor in hippocampal tissue of both WT and homozygous M_1_-mEGFP–expressing animals was in the region of 0.5 to 0.6 pmol/mg membrane protein (Fig. 2*C*). We next assessed whether the presence of mEGFP might interfere with the ability of muscarinic agonism to activate heterotrimeric G proteins. The M_1_-receptor couples selectively to members of the G_q_/G_11_ G protein subfamily (16, 17). We therefore assessed both basal and agonist-promoted activation of the binding of [^35^S]GTPγS in combined hippocampal and cortical membrane preparations. In such studies, it was necessary to enrich [^35^S]GTPγS bound to G_αq_/G_α11_ G proteins by immunoprecipitation of these subunits at assay termination (17–19). In samples prepared from WT mice, the synthetic acetylcholine mimetic carbachol produced a substantial, approximately threefold, increase in bound [^35^S]GTPγS (Fig. 2*D*). This was not different in samples processed from homozygous M_1_-mEGFP–expressing mice (Fig. 2*D*). M_1_ was clearly the muscarinic receptor subtype responsible for this effect, as carbachol was unable to promote binding of [^35^S]GTPγS in equivalent G_αq_/G_α11_ immunoprecipitated samples generated from M_1_-KO mice (Fig. 2*D*). To define that the M_1_-mEGFP construct was fully able to induce more complex functional responses in the animals, we assessed locomotion of homozygous M_1_-mEGFP–expressing mice and compared this with both WT and M_1_-KO animals (*SI Appendix*, Fig. 2). It has previously been established that in this genetic background, loss of function of the M_1_ receptor results in enhanced locomotion in open-field tests (20). Indeed, also here, compared with WT mice, M_1_-KO animals were substantially more active (*P* < 0.001) (*SI Appendix*, Fig. 2). Homozygous M_1_-mEGFP mice, however, displayed no enhanced movement compared with WT mice (*SI Appendix*, Fig. 2).

**Table 1.** Muscarinic receptor expression levels are not different in M_1_-mEGFP and WT mice

		B_max_ (fmol/mg)	K_d_ (nM)
Cortex	M_1_-WT	1,098 ± 353	0.50 ± 0.32
	M_1_-mEGFP	1,203 ± 291	0.56 ± 0.40
	M_1_-KO	582 ± 84	0.35 ± 0.24
Hippocampus	M_1_-WT	989 ± 244	0.54 ± 0.29
	M_1_- mEGFP	783 ± 177	0.88 ± 0.30
	M_1_-KO	426 ± 112	0.46 ± 0.18

Specific binding of [^3^H]NMS was used to quantify total muscarinic-receptor subtype levels in cortex and hippocampus from WT, homozygous M_1_-mEGFP and M_1_-knockout (M_1_-KO) mice. Data are reported as mean ± SD from five animals. One-way ANOVA indicated no significant difference between WT and M_1_-mEGFP in terms of both B_max_ (maximum specific binding) and the K_d_. Values from M_1_-KO mice allowed the contribution of the M_1_-receptor subtype to be assessed (see text for details), with the assumption that elimination of the M_1_ receptor does not substantially alter the expression of other muscarinic-receptor subtypes.

**Fig. 3.**
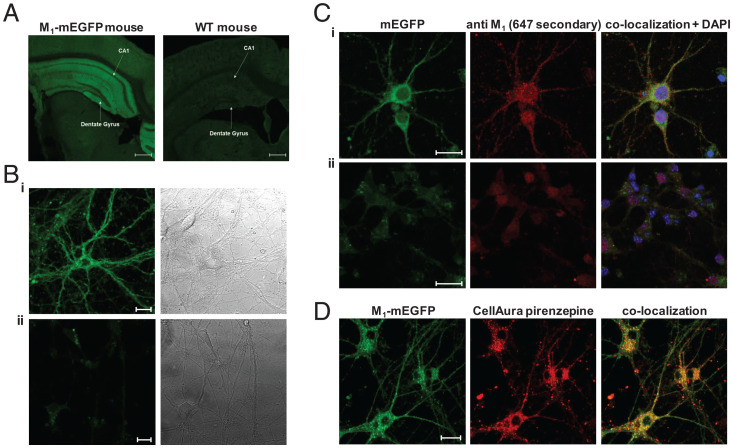
Images of M_1_-mEGFP expression. Fluorescence imaging of brain slices from homozygous M_1_-mEGFP and WT mice shows the profile of expression of M_1_-mEGFP. (*A*) The hippocampal CA1 region and the dentate gyrus are noted. Endogenous fluorescence of the mEGFP tag in the hippocampus is preserved following fixation. (*B*) Images of cultures of (*B*, *i*) M_1_-mEGFP–expressing or (*B*, *ii*) M_1_-knockout (KO) neurons (*Left*) with corresponding bright-field image (*Right*). (*C*) Immunostaining of such neurons from M_1_-mEGFP–expressing (*C*, *i*) or M_1_-KO (*C*, *ii*) neurons with an anti-M_1_ receptor antiserum shows strong colocalization with mEGFP fluorescence in *C*, *i* but not *C*, *ii*. DAPI (blue) provides nuclear staining. (*D*) Addition to such neurons of a fluorescent form of the M_1_ selective antagonist pirenzepine (100 nM; red) also shows strong colocalization with mEGFP fluorescence. Scale bars, 500 µm.

To prepare for M_1_-receptor quaternary structure analysis, we initially imaged slices of brain from homozygous M_1_-mEGFP mice and compared these with equivalent tissue from WT mice. Hippocampal regions showed expression corresponding to M_1_-mEGFP that was lacking in tissue slices from WT animals (Fig. 3*A*). We next isolated cortico-hippocampal neurons from day 16 embryos of homozygous M_1_-mEGFP and M_1_-KO mice and maintained these in culture for 7 d (DIV7). Imaging of such preparations showed M_1_-mEGFP was widely distributed in individual cells across both the cell body and various projections (Fig. 3 *B*, *i*). This, once again, represented M_1_-mEGFP, because no such pattern was observed in cells isolated from the M_1_-KO animals (3 *B*, *ii*). Moreover, immunostaining of such cells with the anti–M_1_ receptor antiserum showed strong overlap with the fluorescent signal of mEGFP (Fig. 3 C, *i*) in cells from the M_1_-mEGFP–expressing line, but not from M_1_-KO mice (Fig. 3 *C*, *ii*), while colabeling of cells with a fluorescent analog of the M_1_-selective antagonist pirenzepine also provided strong overlap of distribution with the mEGFP signal (Fig. 3*D*). Examination of such images suggested that a substantial proportion of the receptor was located intracellularly and that the internalized receptors were not distributed evenly throughout the cell bodies and projections (Fig. 3 *B*–*D*). To explore this in more detail, we labeled such neuronal cultures, from M_1_-mEGFP–expressing mice, with a plasma membrane–marking MemBright dye and then imaged them. This allowed generation of pseudo–three-dimensional images from a series of z-scans while, in addition, labeling in parallel with Hoechst 33342–defined cell nuclei (*SI Appendix*, Fig. 3). Not all cells in the culture were positive for M_1_-mEGFP (*SI Appendix*, Fig. 3). This was not surprising as the culture was derived simply from hippocampal and cortical regions of the day 16 embryo brain, while all such cells and their projections were labeled by the MemBright dye (*SI Appendix*, Fig. 3). However, these studies confirmed that a proportion of M_1_-mEGFP was at the plasma membrane of identified neurons as defined by colocalization with the MemBright dye (*SI Appendix*, Fig. 3) and that a proportion of M_1_-mEGFP was indeed intracellular as it did not colocalize with the MemBright dye (*SI Appendix*, Fig. 3).

**Fig. 4.**
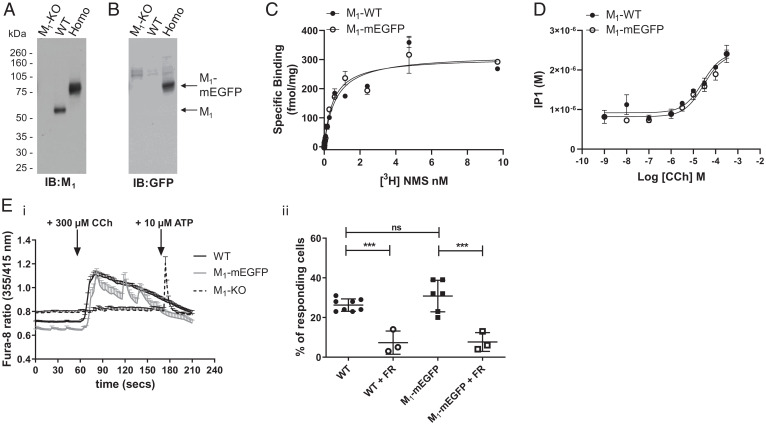
Expression and function of M_1_-mEGFP in neuronal cultures. Lysates from primary neuronal cells generated from day 16 embryos of WT, M_1_-mEGFP homozygous (Homo) knock-in and M_1_-knockout (KO) mice were resolved by SDS-PAGE and immunoblotted with either anti-M_1_ (*A*) or anti-GFP (*B*) antisera. (*C*) Specific binding of varying concentrations of [^3^H]NMS to intact neurons from such cultures of WT and homozygous M_1_-mEGFP–expressing mice was measured to allow assessment of both B_max_ (maximum specific binding) and K_d_ for the ligand. Neither parameter was significantly different (*P* > 0.05) for the two sets of neurons. A representative example of three experiments is shown with error bars representing the SD (*D*). Such neuronal cultures from WT or homozygous M_1_-mEGFP–expressing mice were used to measure the production of inositol monophosphates in response to varying concentrations of carbachol (CCh). Cultures of combined hippocampal and cortical neurons from WT, homozygous M_1_-mEGFP–expressing and M_1_-KO mice maintained for 7 d were loaded with Fura-8-AM. (*E*, *i*) These were then imaged over time in the absence of or after exposure to 300 μM carbachol. In cells isolated from M_1_-KO mice, after exposure to carbachol, 10 μM adenosine triphosphate (ATP) was added to confirm that the cells were able to respond to an external stimulus. (*E*, *ii*) In certain experiments, cells were pretreated with the G_q_/G_11_ inhibitor FR900359 (FR) (21). Data, shown as a percentage of cells tested that responded to carbachol are results taken from three to eight individual mice with analysis of between 31 and 93 cells from each animal. ***, *P* < 0.001. ns, not significantly different.

Immunoblotting with the same M_1_-receptor antiserum used earlier after generation of lysates of such neuronal cultures taken from M_1_-KO, WT, and homozygous M_1_-mEGFP mice and their resolution by SDS-PAGE again showed expression of the 55-kDa WT receptor and the 80-kDa M_1_-mEGFP polypeptide only in the anticipated samples (Fig. 4*A*). Moreover, immunoblotting with the anti-GFP antiserum identified the 80-kDa species only in cultures from M_1_-mEGFP–expressing mice (Fig. 4*B*). Direct measures of specific binding of [^3^H]NMS to intact neuronal cultures confirmed equivalent expression levels in cells isolated from WT and homozygous M_1_-mEGFP–expressing lines (Fig. 4*C*) and that the dissociation constant (K_d_) of the radioligand (K_d_ for WT mice = 0.76 ± 0.22 nM and M_1_-mEGFP mice = 0.74 ± 0.19 nM) was not different between these preparations (Fig. 4*C*). Addition of carbachol to such neuronal cultures promoted production of inositol phosphates in a concentration-dependent manner. This was also indistinguishable between neurons derived from WT and homozygous M_1_-mEGFP mice (pEC_50_ [the negative log of the half maximal effective concentration] for WT mice = 4.62 ± 0.04 and M_1_-mEGFP mice = 4.59 ± 0.30) (Fig. 4*D*). As expected from agonist-induced production of inositol phosphates, carbachol promoted elevation of [Ca^2+^], and these were very similar in neuronal cultures from WT and homozygous M_1_-mEGFP–expressing mice both in extent and time course (Fig. 4 E, *i*). By contrast, carbachol did not promote a large increase of [Ca^2+^] in cells isolated from M_1_-KO mice, although subsequent addition of the P2 purinoceptor agonist adenosine triphosphate was able to elicit rapid elevation of intracellular [Ca^2+^] (Fig. 4 *E*, *i*). This effect of carbachol was transduced via G_q_/G_11_ G proteins because elevation of [Ca^2+^] was prevented (*P* < 0.001) by pretreatment with the highly selective G_q_/G_11_ inhibitor FR900359 (21) (Fig. 4 *E*, *ii*). These outcomes are consistent with release of Ca^2+^ from inositol 1,4,5 trisphosphate–sensitive intracellular stores. Consistent with only a subset of the cultured neurons expressing M_1_-mEGFP, only some 36.7% ± 4.0% (mean ± standard deviation [SD]) of tested cells assessed from these animals responded to carbachol, and this proportion was similar to the percentage of cells from WT mice (26.8% ± 3.4%) (Fig. 4 *E*, *ii*). Additionally, addition of carbachol promoted phosphorylation of M_1_-mEFGP at residue Ser^228^ (*SI Appendix*, Fig. 4), as has previously been shown for the WT and other modified forms of the receptor in both cell lines and in mice (22).

**Fig. 5.**
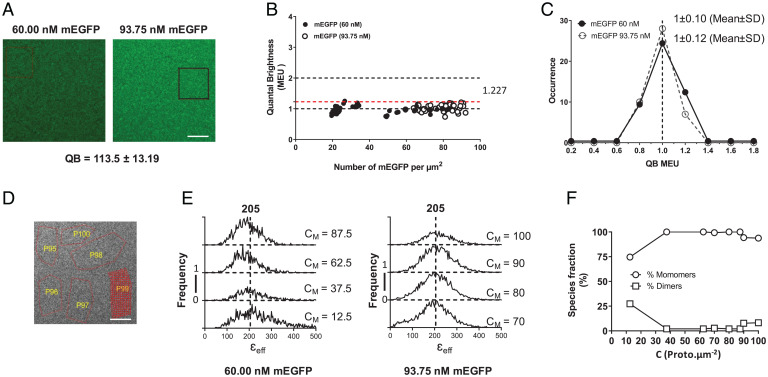
SpIDA and FIF spectrometry define the QB and monomeric state of mEGFP. Solutions of 60 nM and 93.75 nM mEGFP were imaged and analyzed by SpIDA. A total of 45 RoI were assessed for each concentration. (*A*) The highlighted box in the 93.75-nM image shows representative RoI. Scale bar, 20 µm. These provided QB values of 113.5 ± 13.2 (mean ± SD) for mEGFP. (*B* and *C*) SpIDA indicated that mEGFP is monomeric across all concentrations (*B*) (the dotted line = 1.227 is the median QB [designed as 1.00 MEU + 1.96 SD above MEU, which corresponds to 95% of the observations]) and that data were normally distributed around the MEU (*C*) (see refs. 13 and 43 for details). (*D*–*F*) FIF spectrometry was conducted on segmented elements (red; P99 in the image) of randomly selected RoIs (red outlines, P95 to P100) (*D*). Scale bar, 20 µm. We characterized 38 RoIs for 60.0 nM and 100 RoIs for 93.75 nM. (*E* and *F*) Analysis determined a monomeric brightness of 205 and confirmed that across the range of concentrations, mEGFP was monomeric apart from values at very low density (mean, 12.5 molecules/μm^2^) (see ref. 7 for full details). See text for further details of why analyses of RoIs with receptor density of fewer than 15 molecules/μm^2^ was excluded from the final data set. C_M_ is the area concentration of the membrane in units of of protomers (proto).μm^−2^.

Recently, methods based on FFA have been employed to assess quaternary organization of fluorophore-tagged transmembrane proteins in a range of settings (5–7). It is vitally important for demonstration of the existence and quantification of the proportion of such complexes to define fluorophore quantal brightness (QB) for a monomeric standard protein at concentrations and levels close to those of the protein of interest. To do so, we employed solutions of mEGFP at 60 nM and 90 nM. Spatial intensity distribution analysis (SpIDA) (10, 23–25) performed on images of such preparations provided QB of 113.5 ± 13.2 (mean ± SD) (Fig. 5*A*). Such SpIDA studies indicated that across levels between 20 and 90 molecules/µm^2^ mEGFP was entirely monomeric (Fig. 5*C*). Segmentation of RoIs in such images (Fig. 5*D*) and the application of FIF spectrometry (7–10) confirmed that across levels ranging from 20 to 100 molecules/µm^2^, mEGFP was entirely monomeric (Fig. 5 *E* and *F*).

**Fig. 6.**
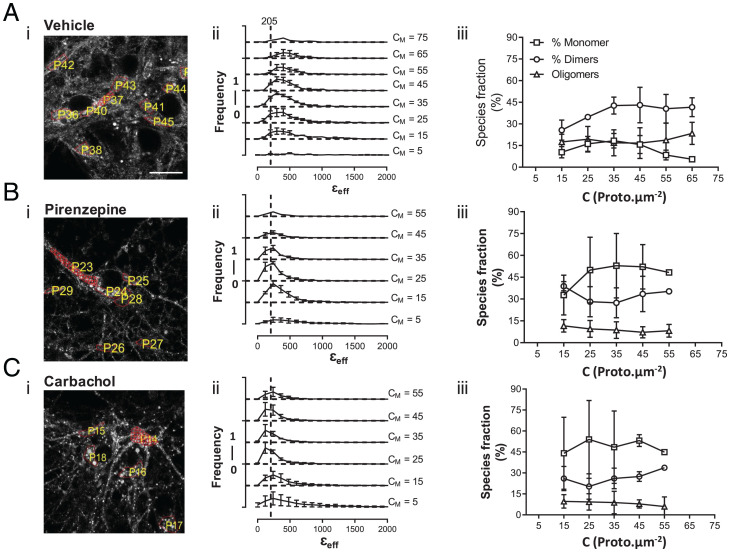
Quaternary organization of the M_1_ receptor in hippocampal cortical neuronal cultures from M_1_-mEGFP–expressing mice. (*A*–*C*) M_1_-mEGFP–expressing neuronal cultures maintained without ligand (*A*) or after treatment with pirenzepine (16 h, 1 × 10^−5^ M) (*B*) or carbachol (30 min, 1 × 10^−3^ M) (*C*), were imaged; RoIs were selected as described in Fig. 5 (illustrative RoIs are numbered), and segmented (red) (*A*, *i*, *B*, *i*, and *C*, *i*) to allow application (*A*, *ii*, *B*, *ii*, and *C*, *ii*) and quantification (*A*, *iii*, *B*, *iii*, and *C*, *iii*) of quaternary organization structure via FIF spectrometry. Data derive from three separate neuronal preparations in which 62, 70, and 54 (total = 186) (*A*); 100, 113, and 150 (total = 363) (*B*); and 113, 100, and 100 (total = 313) (*C*) RoIs were examined. (*A*, *ii*, *B*, *ii*, and *C*, *ii*) The density of M_1_-mEGFP varied within different segmented RoIs. The dotted line labeled 205 indicates the peak ε_eff_ anticipated for a pure population of monomers. (*A*, *iii*, *B*, *iii*, and *C*, *iii*) Proportions of monomers, dimers, and oligomers of M_1_-mEGFP were estimated for distinct receptor density bins and plotted as a function of receptor concentration. Data are reported as mean ± SD. As mole fractions of these species were not significantly different over the range of 25 to 55 molecules/μm^2^, all data from this concentration range were combined to produce the outcomes shown in Fig. 7. Scale bar, 20 μm. C_M_ is the area concentration of the membrane in units of of protomers (proto).μm^−2^.

**Fig. 7.**
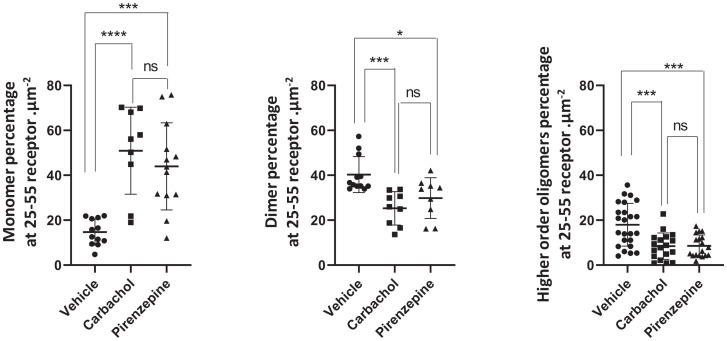
Both the agonist carbachol and the antagonist pirenzepine favor monomerization of M_1_ in neuronal cultures of M_1_-mEGFP–expressing mice. Results from FIF spectrometry analysis of neuronal cultures from M_1_-mEGFP–expressing mice as detailed in Fig. 6 are presented as monomers, dimers, and oligomers for data sets encompassing receptor density across the range of 25 to 55 molecules/μm^2^. *P < 0.05, ***P < 0.001, ****P < 0.0001. One way ANOVA followed by Tukey’s multiple comparisons test. ns, not significant.

We have used SpIDA extensively to assess GPCR quaternary structure in transfected cell lines in culture and in settings in which the receptor is relatively equally distributed in the cell plasma membrane (10, 23–25). However, as noted earlier, in neuronal cultures taken from the transgenic M_1_-mEGFP–expressing mice, the pattern and distribution of the receptor were more complex, with plasma membrane delineated and intracellular components of varying intensity. FFA analysis based on approaches including SpIDA, number and brightness analysis, and photon-counting histogram have marked limitations for more complex and real tissue-based studies because they tend to average out oligomer size from potentially complex mixtures of oligomers of different sizes in defined and imaged RoIs (7, 8). Thus, we employed FIF spectrometry (7–10) to analyze the organizational state of the M_1_-mEGFP in neuronal cultures from M_1_-mEGFP–expressing mice (Fig. 6). We determined the molecular brightness of mEGFP molecules to be 205 arbitrary units (a.u.) by imaging a solution of mEGFP but focusing the laser beam on the interface between the coverslip and the solution liquid. Because, at the interface, most of the fluorescence signal emanates from a thin layer of mEGFP molecules adsorbed nonspecifically to the coverslip, we used a γ value of 0.5 for the laser beam shape factor, which compensates for the nonuniform intensity distribution of the laser power across the molecules within the focal volume. Fortunately, this is the same value of the γ factor, which is found for all membrane orientations imaged using a confocal microscope with a pinhole size of 1 Airy unit (see *γ Factor Calculation* in *Materials and Methods*). In defined and segmented RoIs covering both membrane and intracellular locations (Fig. 6), we observed that receptor density ranged from some 5 to 65 promoters/µm^2^ and, importantly, was thus largely within the calibration range established for mEGFP of between 20 and 100 promoters/µm^2^ (Fig. 5). In the basal state, FIF spectrometry–based extraction of oligomeric-state information for M_1_-mEGFP indicated that monomers were not the most abundant form, as indicated by peak brightness (ε_eff_) values being consistently located at values higher than anticipated for a monomer (see dotted line at ε_eff_ =205 in Fig. 6 *A*, *ii*) and, that as well as a substantial fraction of dimers (mean ± SD, 40.3% ± 8.0%), there was also a marked proportion (mean ± SD, 17.9% ± 9.5%) of oligomeric species (Fig. 6*A*). Observations from RoIs with a mean concentration of 15 promoters/µm^2^ or less were sparse, as were those from RoIs with mean concentrations greater than 55 promoters/µm^2^. Because the estimated proportions of different-sized complexes did not vary markedly over the central expression range of 25 to 55 promoters/µm^2^ (Fig. 6*A*) and that definition of mEGFP as a monomer was not valid at density of fewer than 15 molecules/µm^2^ (Fig. 5*F*), we focused analysis by combining data sets across the range of 25 to 55 promoters/µm^2^ (Figs. 6 and 7), where monomers composed, on average, 14.7% ± 5.8% (mean ± SD) of the population (Fig. 7).

An intriguing question was whether the proportions of monomers, dimers, and oligomers of M_1_-mEGFP in such neurons might be altered by exposure to muscarinic ligands. Initially, we assessed the effect of the M_1_ selective antagonist pirenzepine. At a gross observational level, treatment of the neuronal cultures with pirenzepine (1 × 10^−5^ M; 16 h) at a concentration predicted to occupy more than 98% of the receptors did not alter the distribution of M_1_-mEGFP (Fig. 6*B*). However, FIF spectrometry analysis showed that with such exposure to pirenzepine, the proportion of monomers increased markedly (*P* < 0.001) to now account for some 44.0% ± 5.4% of the receptor population. This was accompanied by a statistically significant reduction (*P* < 0.05) in the proportion of dimers and, in addition, a marked reduction in the proportion of oligomeric complexes (*P* < 0.001) (Fig. 7). Pretreatment with carbachol (1 × 10^−3^ M; 30 min) increased the apparent punctate distribution of the receptor (Fig. 6*C*), potentially linked to enhanced internalization, and also markedly increased the proportion of monomeric species (*P* < 0.0001), again with an associated reduction in the dimer (*P* < 0.001) and oligomer (*P* < 0.001) fractions (Fig. 7). To test whether the apparent increase in the proportion of monomeric species after treatment with carbachol instead could be attributed to the presence of punctae in the images, we implemented an automated procedure (9) to remove the high-intensity spots (attributed to internalized vesicles) from the fluorescence images. We then performed the FIF analysis on the “despotted” fluorescence images and, once again, found an increase in the proportion of monomeric species when compared with the WT images. This analysis confirmed that the apparent increase in relative monomer abundance as a result of carbachol treatment was, indeed, real and not an artifact associated with the increased density of the vesicle-like spots in the fluorescence images. It should be noted, however, that the maximum-brightness spectrogram for carbachol-treated samples (Fig. 6 *C*, *ii*) falls somewhat below the expected monomeric brightness indicated by the dashed vertical line. This may be caused by an inadvertent increase in the γ factor due to reduced membrane folding following loss of membrane through generation of endocytic vesicles in this sample. This may contribute to the apparent decrease in oligomer size for agonist-treated receptors.

## Discussion

The extent of quaternary organization of class A GPCRs, other than rhodopsin, has been questioned for many years (1, 26–28). Despite the development and use of a wide range of methods, and these being directed at a substantial number of different receptors (29, 30), no clear consensus has emerged. In part, this has reflected that, in a cellular setting, most of the studies have been performed using simple heterologous cell lines and have frequently relied on transient transfection and expression of constructs of interest. This has meant that receptor expression levels have not been reported in a systematic manner and that there has been a lack of reproducibility between studies even when these have employed the same GPCR. Despite early studies reaching varying conclusions, it is now clear that GPCRs can interact, and that this can be observed to occur in a concentration- and sometimes ligand-dependent manner (31).

We wished to assess the topic of the extent of quaternary structure of a central nervous system–expressed GPCR and selected the M_1_ muscarinic receptor as an exemplar because it is known to be expressed at good levels in the hippocampus and cortex and because it is a therapeutic target where activation is viewed as a strong potential therapeutic modality (32). There are numerous ways in which protein–protein homomeric interactions could be explored in native cells. However, we considered the positive of FFA only requiring a single fluorophore-tagged protein as paramount. Although other GPCR–fluorescent protein (FP) knock-in mouse models have been produced (33–37), these have not been used to address similar questions as we did in the present study, possibly in part because they did not employ mutationally modified forms of the FP designed to limit self-association. A specific feature of the construct we employ is the (Ala^206^Lys) mutation of EGFP that greatly reduces the propensity of the FP to self-associate (14). Without this specific variant, which we show to be entirely monomeric over the expression range of the M_1_-mEGFP construct in neurons cultured from the transgenic line, it would have been impossible to separate the propensity for self-association of the GPCR from effects produced by the fluorophore. Moreover, we have recently developed the approach described as FIF spectrometry (7–10) that has overcome issues of smoothing out of quaternary structure complexity that is inherent in other FFA-based methods. This capacity has previously proved of great value in demonstrating the complexity of quaternary organization of both the epidermal growth factor receptor tyrosine kinase and the secretin receptor, a class B GPCR, when expressed at supraphysiological levels in Chinese hamster ovary cells (7–9). In addition, FIF possesses a built-in filter for regions that present much higher fluorescence intensities than the surrounding plasma membrane, such as coated pits and endocytic vesicles (7–9). Thus, despite the complexity of varying levels of expression of M_1_-mEGFP in different locations of neurons taken from the knock-in M_1_-mEGFP–expressing mice, and the fact that a substantial proportion of the receptor was present within punctate intracellular locations rather than being restricted to the plasma membrane, we have been able to detect and quantify each of monomeric, dimeric, and even oligomeric (potentially trimeric and tetrameric) forms of the receptor in assessed RoIs of these cells. Analysis was made across a relatively small range of receptor expression levels because the majority of interrogated RoIs expressed M_1_-mEGFP at levels between 20 and 60 molecules/µm^2^. While studies in transfected cell systems have shown a relationship between receptor density and quaternary complexity, it is perhaps not surprising that over this rather limited expression range, we were unable to observe a clear relationship. We thus combined data sets from RoIs over this expression range to provide greater statistical power in defining whether the binding of agonist or antagonist ligands might drive differences in the proportion of the distinct quaternary complexes. Since FIF spectrometry, and indeed any FFA-based method, cannot resolve distances between protomers within an oligomer, one cannot exclude the possibility that the association of the M_1_ receptors is mediated by other cellular components, such as other proteins interposed between protomers or larger structures such as coated pits within which the receptors are trapped before their internalization. Nevertheless, results of our previous studies using Förster resonance energy transfer (FRET) on other muscarinic receptor subtypes (38, 39) indicated that the receptors are within touching distances of one another inside the oligomers.

Treatment of neuronal cultures for a sustained period with a concentration of the M_1_-selective antagonist pirenzepine calculated to be sufficient to occupy greater than 98% of the receptors caused a substantial alteration in the overall makeup and oligomeric distribution of the receptor population, with marked enrichment of the proportion of monomers and a decline in the dimeric fraction. The topic of whether antagonist ligands generally affect receptor quaternary organization is a complex one. In transfected cell systems, pirenzepine has previously been reported to increase the dimeric and oligomeric fraction of the M_1_ receptor (11, 13). In contrast, although the antipsychotics spiperone and haloperidol have been reported to destabilize dimers of the D_3_ dopamine receptor, various other blockers of this receptor did not have this effect (40). Moreover, while the antagonist ligand IT1t has been shown to disrupt dimers of the chemokine CXCR4 in multiple independent studies (10, 41), other antagonists of this receptor do not produce such an effect (41). There are, hence, many current unknowns. Despite many efforts to employ informatic analysis to define the most likely basis for class A GPCR dimerization (e.g., see refs. 42, 43, and 44), it has been challenging to define common rules. Interestingly we also were able to show that the agonist ligand carbachol favored monomerization of the receptor, even in the context of the agonist promoting further internalization of the receptor. This is of considerable interest as recent studies have suggested that activation of this receptor with a selective agonist may be beneficial in maintaining cognitive functions in both mouse models and patients suffering from neurodegenerative conditions (32). The observations that both an agonist and an antagonist ligand had similar effects on the oligomeric state of this receptor may at first seem surprising, as they are often, incorrectly, viewed simply as producing opposite effects. However, carbachol and pirenzepine are both orthosteric ligands binding to overlapping sites on the receptor and this may be the key trigger. On the other hand, it is possible that some of the decrease in oligomer size triggered by treatment with carbachol may be explained by an increase in membrane smoothness due to loss of membrane via generation of endocytic vesicles (see *Results*). It will be interesting to explore how allosteric ligands at this receptor may affect these quaternary complexes.

In these studies, we observed and quantified the quaternary organization of a class A GPCR in native tissue without resorting to overexpression. The M_1_-receptor in these neurons is expressed at only 0.1% to 0.3% of the density of rhodopsin; hence, the methods used herein should be amenable to studying other central nervous system–expressed GPCRs that are present at significant levels, including those for opioids, cannabinoids, and dopaminergic ligands that are the targets of both therapeutically important medicines and drugs of abuse. They should also be equally well suited to study other transmembrane proteins, including neurotransmitter transporters. Clearly, methods such as FRET and bioluminescence resonance energy transfer that require the co-expression of both energy donor and energy acceptors fused to the protein of interest are poorly suited for such studies, but methods based on FFA, specifically FIF spectrometry, are, as shown here, entirely capable of defining protein quaternary structure at native expression levels. Rapidly developing methods in genome editing, as well as more traditional approaches to gene knock-in, will now allow such questions to be addressed widely. Of course, the addition of an FP to a receptor clearly requires that careful studies define the functional equivalence and potential effects on expression level of the transgene prior to initiating a more detailed analysis. There is also the potential to employ genome-editing methods to introduce an appropriate fluorophore into GPCRs or other transmembrane proteins expressed endogenously by various cell lines that are widely used in biomedical research. These may also allow other methods to be developed, adapted, and quantified. As native expression levels of many class A GPCRs are markedly lower than the M_1_ receptor we have used as an exemplar here, these may require the use of brighter and smaller fluorophores than mEGFP. Although we demonstrate here that class A GPCR quaternary organization is a reality in native tissue and that it is regulated, at least for this receptor, by ligand occupancy, the ongoing challenge will be to demonstrate categorically its significance for function and then for the therapeutic targeting of such receptors (1, 2, 31).

## Materials and Methods

### Animals.

M_1_-mEGFP transgenic knock-in mice on the C57BL/6N background were generated by GenOway. A loxP-stop-loxP cassette containing M_1_-mEGFP was inserted into the *Chrm1* endogenous locus. Embryonic stem cells were introduced into blastocysts that were implanted in pseudopregnant females to generate chimeric mice. Such chimeric mice were bred with Cre-recombinase–expressing mice to produce animals in which M_1_-mEGFP replaced the M_1_ receptor.

#### Genotyping.

We used the following primers predicted to generate a single band of 223 bp (WT) or a single band of 332 bp (homozygous M_1_-mEGFP): 168766seq-TOB18 common primer 5′ TGG TGC CAG GAC GGT GAT GTT G; 9206cre-TOB18 5′ ACA TGG TAA GTA AGC TTG GGC TGC AGG; and 9208cre-TOB18 5′ CTG GCC TGG CAC TCT GAA AGG TCT. As such, heterozygous animals were anticipated to generate both these products 223 bp and 332 bp.

#### Animal maintenance.

WT C57BL/6 mice, M_1_-mEGFP transgenic knock-in and M_1_ knock-out mice, also on the C57BL/6N background, were fed ad libitum with a standard mouse chow diet. Male and female animals at 8 to 15 wk old were used for timed matings. Animals were cared for in accordance with national guidelines on animal experimentation. All animal experiments were conducted under appropriate home office licenses. Experiments were conducted under project establishment license number 70/8473.

### Materials.

Horseradish peroxidase–linked rabbit anti–goat IgG, poly-d-lysine and complete protease and phosphatase inhibitors mixture were from Sigma-Aldrich. Horseradish peroxidase–linked sheep anti-mouse was from Abcam. Alexa Fluor 647 anti-mouse was from ThermoFisher. Anti-GFP antiserum was generated in-house. Anti-M_1_ antibody [mAChR M_1_ Antibody (G-9), sc-365966] was from Santa Cruz. Anti-pSer^228^ M_1_ was generated in-house in collaboration with Eurogentec (22). ECL reagent was purchased from Pierce. [^3^H]NMS, [^35^S]GTPγS, and Microscint-20 were from Perkin-Elmer. CellAura fluorescent pirenzepine was from Hello Bio Ltd.

B-27 plus neuronal culture system; TripLE Select 10×; diethyl pyrocarbonate–treated water;, Hank’s Balanced Salt Solution (HBSS); penicillin-streptomycin; l-glutamine; Opti-MEM I Reduced Serum Media; lipofectamine 2000; laminin mouse protein; NuPage Novex precast 4% to 12% Bis-Tris gels; NuPage MOPS SDS running buffer; and both 4′,6-diamidino-2-phenylindole (DAPI) and Hoechst 33342 stains were from Life Technologies. Fura-8 AM was purchased from Stratech Scientific Limited. Lipilight MemBright 640 was from Idylle.

### Methods.

#### Plates and cover-slides coating.

Tissue culture plates and cover slides were coated as described previously (45).

#### Primary neuronal culture.

The hippocampal and cortical areas of the brain were isolated from E16 embryos and primary culture carried out as described (45).

#### Protein extraction from brain tissue.

Brain tissues were homogenized and protein extracted as described by Scarpa et al. (45).

#### Lysates from Flp-In TREx 293 M_1_-mEGFP stable cells and from primary neuronal cells.

Cells were harvested in ice-cold phosphate-buffered saline (PBS; 120 mM NaCl, 25 mM KCl, 10 mM Na_2_HPO_4_, and 3 mM KH_2_PO_4_, at pH7.4) and lysed in radioimmunoprecipitation assay buffer (supplemented with protease and phosphatase inhibitors) on a rotating wheel for 30 min at 4 °C. Samples were then centrifuged for 15 min at 21,000 × *g* at 4 °C, and the supernatant aliquoted and stored at −80 °C until required.

### M_1_-mEGFP Receptor Immunoprecipitation and Immunoblotting Assays.

The mEGFP-linked receptor construct was immunoprecipitated from 540 µL of cell lysate (3 µg/µL protein) using the GFP-Trap kit (Chromotek) according to manufacturer's instructions. Immune complexes were washed three times in washing buffer, resuspended in 100 µL of 2× SDS-PAGE sample buffer and incubated at 60 °C for 10 min. Following centrifugation at 2,500 × *g* for 5 min, 40 µL of immunoprecipitated proteins were resolved by SDS-PAGE on 4% to 12% BisTris gels. After separation, immunoblots were carried out as described by Marsango et al. (40), with the following modifications: Nitrocellulose membranes were blocked using 5% bovine serum albumin (BSA) in Tris-buffered saline (50 mM Tris-Cl, and 150 mM NaCl, at pH 7.6), and anti-pSer^228^ M_1_ primary and anti-rabbit secondary antibodies were diluted 1:1,000 and 1:10,000, respectively, in 5% BSA in Tris-buffered saline supplemented with 0.1% Tween.

#### Immunoblots.

Lysate from brain tissue or primary neuronal cells prepared as described above were diluted to a final concentration of 1 mg/mL in lysis buffer. Samples were prepared by the addition of SDS-PAGE sample buffer and heated to 55 °C for 5 min. Equal amounts of protein (15 µg) from each sample were loaded into wells of 4% to 12% BisTris gels and subjected to SDS-PAGE analysis using NuPAGE MOPS SDS running buffer. After separation, immunoblots were carried out as described (40). Anti–GFP antiserum was diluted 1:10,000, anti-M_1_ antiserum diluted 1:500, secondary antisera (horseradish peroxidase–linked rabbit anti–goat immunoglobulin G or horseradish peroxidase–linked sheep anti-mouse immunoglobulin G) were diluted 1:10,000.

#### [^3^H]NMS-binding studies on membrane preparations.

We performed [^3^H]NMS-binding studies on membrane preparations as described previously (20).

#### [^3^H]NMS binding studies on intact primary neuronal cells.

Primary neuronal cells were seeded at a density of 5 × 10^4^ cells/well onto a 96-well plate and maintained at 37 °C in a 5% CO_2_ humidified atmosphere. On the sixth day in vitro, cells were washed twice with binding buffer (110 mM NaCl, 5.4 mM KCl, 1.8 mM CaCl_2_, 1 mM MgSO_4_, 25 mM glucose, 20 mM 4-(2-hydroxyethyl)-1-piperazineethanesulfonic acid [HEPES], 58 mM sucrose). A range of [^3^H]NMS concentrations (between 0 and 10 nM) were added to appropriate wells containing buffer or 10-µM atropine to determine total and nonspecific binding in 100 µL of final-volume binding buffer. Plates were incubated for 2 h at 37 °C and the reactions were terminated by removal of the binding mixture followed by 3 × 200 µL/well ice-cold NaCl 0.9% washes. We added 100 µL/well of Microscint-20, and the plates were sealed before overnight incubation at room temperature on a rapidly shaking platform. Bound ligand was determined using a Packard Topcount NXT (Perkin-Elmer Life Sciences).

#### Imaging hippocampal slices.

For sample acquisition, mice were transcardially perfused with 10 mL of 9.25% sucrose in phosphate buffer, followed by 40 mL of 4% paraformaldehyde. Samples were then postfixed in 4% paraformaldehyde at 4 °C for 24 h, washed in phosphate buffer, and cryoprotected in 30% sucrose until saturated. Then, 1-mm thick tissue blocks containing the hippocampus were embedded in optimal cutting temperature compound and sectioned using a Leica CM1860 UV Cryostat. Sections were mounted on slides washed in PBS and mounted using Vectashield Mounting Medium containing DAPI. Images were acquired on a confocal microscope at a ×10 magnification.

#### Imaging primary neuronal cells.

Primary neuronal cells were seeded at a density of 5 × 10^5^ cells onto precoated, 30-mm cover slides and maintained at 37 °C in a 5% CO_2_ humidified atmosphere. On DIV7, cells were washed three times with HBSS and imaged using a Zeiss LSM 880 confocal equipped with a ×63/1.4 numerical aperture (NA) plan apochromat oil-immersion objective. The 488-nm argon laser line was used to sequentially excite mEGFP.

#### Confocal plasma membrane imaging using MemBright-640 dye.

DIV7 primary neuronal cells cultured on precoated cover-glass slides were washed twice with HBSS and incubated with freshly prepared 100 nM MemBright solution for 10 min at 37 °C. Dye was excited using 633-nm laser light passed through a ×63 plan apochromat oil-immersion objective lens (NA = 1.4). Resultant emission light was detected using a spectral detector set to detect light over the wavelength range of 660 to 700 nm. Gain applied to the photomultiplier tube (PMT) was 587 V.

The confocal microscope *z*-axis stepper motor was utilized to simultaneously acquire multichannel images for three-dimensional (3D) visualization of nuclei and M_1_ receptors located within the membrane (image format, 512 × 512; *x*, *y*, and *z* resolution = 0.22 µm). The individual z-stack images were corrected for *x* and *y* translation before they were merged. The merged images were deconvoluted using a 3D, blind, iterative, and constrained algorithm (Autoquant ×3 software, version 3.1.3; Media Cybernetics). Metamorph software (Molecular Devices; version 7.10.4) was used to create a *z*-*x* blended projection image map showing the location of M_1_ receptors within the membrane and around the nucleus.

### Cell Labeling with Fluorescent Analog of the M_1_-Selective Antagonist Pirenzepine.

DIV7 primary neuronal cells cultured on precoated cover-glass slides were treated overnight with 100 nM CellAura fluorescent pirenzepine. Cells were washed three times in HBSS and then imaged using a Zeiss LSM 880 confocal equipped with a ×63/1.4 NA plan apochromat oil-immersion objective using 633-nm excitation and 650-nm emission.

#### Hoechst 33342 staining.

DIV7 primary neuronal cells were incubated with freshly prepared medium containing 10 µg/mL of the nuclear DNA-binding dye Hoechst 33342 for 15 min at 37 °C. Before imaging, cells were washed twice in HBSS.

#### Drug treatments.

In certain studies using FIF analysis, DIV7 primary neuronal cells were treated with 1 mM carbachol for 30 min or 10 µM pirenzepine overnight at 37 °C in a 5% CO_2_ humidified atmosphere. Images were collected in the presence of the appropriate ligand.

#### [^35^S]GTPγS assay.

Mice (age 8 to 12 wk) were humanely killed and cortical tissue was dissected on ice. Membranes were prepared and [^35^S]GTPγS binding performed as described previously (20).

#### Inositol phosphate accumulation assays.

Inositol phosphate accumulation assays were performed as described by Scarpa et al. (45).

#### Ca^2+^ imaging.

Primary neuronal cells were plated onto precoated cover slides at a density of 5 × 10^5^ and maintained at 37 °C in a 5% CO_2_ humidified atmosphere. On DIV7, cells were loaded with the calcium-sensitive dye Fura-8 AM; 3 µM Fura-8 was added to normal growth medium and the cells incubated at 37 °C for 30 min. Cells were then washed and incubated with HBSS with or without 1 µM FR900359 for 30 min. Coverslips were then placed into a microscope chamber containing HBSS and assays were performed as described previously (46).

### Open-Field Studies.

Locomotor activity was assessed using the open-field test, following overnight habituation in the behavioral testing suite. Mice were placed in a clear, Perspex square arena (50 × 50 cm) and activity was tracked for a 10-min period using ANY-maze software.

#### Immunocytochemistry.

Primary neuronal cells were seeded at a density of 5 × 10^5^ cells onto precoated, 30-mm cover slides and maintained at 37 °C in a 5% CO_2_ humidified atmosphere. On DIV7, cells were fixed in 4% PFA for 15 min at room temperature and then washed twice with PBS. Cells were permeabilized with PBS + 0.1% Triton X-100 and blocked for 45 min at room temperature in blocking buffer (PBS, 10% goat serum, and 2% BSA). Cells were incubated overnight at 4 °C with anti-M_1_ antiserum in blocking buffer (1:1,000; Santa Cruz). Subsequently, cells were washed three times with PBS + 0.05% Tween-20 and incubated with secondary antibody Alexa Fluor anti-mouse 647 (1:400; Thermo Fisher) for 2 h at room temperature in blocking buffer. Following three washes, coverslips were mounted in VECTASHIELD Mounting Medium with DAPI (Vector Laboratories).

#### Preparation of concentration-calibrated monomer solutions of mEGFP.

Lab-Tek II 8-well cover-glass chambers, 1.5 thickness, were cleaned by sonicating them in 1 M potassium hydroxide for 15 min. They were then thoroughly rinsed with Milli-Q water and dry sterilized for 30 min under ultravioly light in a class II biosafety cabinet. To reduce adsorption of mEGFP, cleaned chambers were incubated with 1% BSA dissolved in buffered dilution solution containing 20 mM Tris and 50 mM NaCl (adjusted to pH 8.0, using 1 M HCl) for 24 h prior to imaging. On the day of imaging, the BSA was removed from each well and replaced with dilution solution. The dilution solution was then replaced twice more to ensure that all the BSA had been removed. For imaging, mEGFP fluorescent proteins were diluted to 60 or 93.75 nM in the buffer solution (pH 8.0). To each well, 200 µL of mEGFP solution was added, and the samples were imaged using the same laser power, scanning, and detection parameters detailed in the mEGFP confocal-microscopy-imaging section. The concentration-calibrated monomeric solutions of mEGFP were prepared to quantify the QB value of mEGFP and to define the concentration range that this fluorophore was entirely monomeric.

#### Confocal microscopy imaging of mEGFP-monomer calibration solutions using 488-nm laser excitation light.

A Zeiss 880 laser scanning confocal microscope (invert model) equipped with a ×63 plan apochromat oil-immersion lens was used to record high-resolution images with a lateral pixel size of 60 nm. The 488-nm laser spot scan speed was set to a pixel dwell time of 16.48 µs/pixel and emitted fluorescent light was detected using a tunable gallium arsenide phosphide spectral detector using the following detector parameter settings: emission wavelength range, 505 to 605 nm; gain, 850 V; offset, 0; amplifier gain, 1. The pinhole was set to 1.00 Airy unit and the 488-nm laser power intensity was always set to 2% to ensure the illuminated solution was consistently excited with the same amount of incident 488-nm excitation light. The 488-nm laser-beam waist radius size, PMT shot noise, and background autofluorescence signal were quantified as previously detailed (10, 47).

#### SpIDA.

The SpIDA method has been previously described in full detail (10). A brief, informative summary follows.

#### RoI SpIDA.

RoI QB values measured from images recorded from solutions containing different concentrations of mEGFP monomers (60 and 93.75 nM) were statistically assessed using a paired *t* test and found to be nonsignificant. The QB values from each monomer calibration solution were combined to find a mean QB value across all solutions (113.5; see Fig. 5). This mean value was used to create brightness-related monomeric equivalent unit (MEU) values, (RoI QB value/113.5 = MEU value). To show that the mEGFP-monomer calibration solutions were indeed monomeric, frequency distribution curves (MEU bin size = 0.2) were plotted for each quantified MEU value. For each curve generated, the MEU values around the mean displayed a normal symmetrical distribution, and statistical normality tests (D’Agostino and Pearson, Shapiro-Wilk) confirmed the distributions were Gaussian. To distinguish between monomeric and other species, an MEU value of 1.227 (which represents 95% of the data points spread around the mean + 1.96 SD), was set as a boundary to distinguish protein species greater than a monomer.

#### RoI SpiDA mEGFP-monomer solution-concentration quantification.

The SpIDA software program calculates the mean fluorescence intensity for each RoI analyzed. To determine the average mEGFP concentration within each RoI, an apparent number of particles in the beam area was first calculated by dividing the mean fluorescence intensity for an ROI by the monomeric QB as follows:

[1] $$N_{\text{SpIDA}}=\frac{\left[\text{I}\right]}{{\text{Mean}\;\text{QB}}_{\;(\text{calibration}\;\text{solution})}}.$$

Here, [I] represents the RoI mean pixel fluorescence intensity and the denominator represents the mean QB value measured from 90 RoIs drawn on the images acquired from the monomer calibration solutions (60 and 93.75 nM).

The value quantified from Eq. **1** was then used to determine the total protomer concentration (i.e., number of molecules/µm^2^) of mEGFP molecules in solution (see Eq. **2**):

[2] $$C_{\text{correctSpIDA}}=\frac{N_{\text{SpIDA}}\cdot \gamma }{\iint^{\text{​}}\text{PSF}\left(x,y\right)d\mathrm{xdy}},$$

where *γ* represents a shape factor that depends on the shape of the laser point spread function (PSF) and the geometry of the sample (*γ* = 0.5), which compensates for the nonuniformity of the PSF shape of the laser beam spot.

The numerator $\iint^{\text{​}}\text{PSF}\left(x,y\right)d\mathrm{xdy}$ represents the size of the illumination area from which a fluorescence signal of [I] would be generated if all particles in the beam were positioned in the center of the beam and produced a fluorescent signal. The area integral was quantified using the following equation:

[3] $$\iint^{\text{​}}\text{PSF}\left(x,y\right)d\mathrm{xdy}=\frac{1}{2}\pi {\left(w_{xy}\right)}^{2}=0.111\;\text{µ}{\text{m}}^{2},$$

where a value of $w_{xy}=0.2656\;$ was used for the laser-beam waist (measured using 100-nm fluorescent TetraSpeck beads).

#### FIF spectrometry analysis.

FIF spectrometry analysis has been previously described in full detail (7–10, 48). FIF measures the population fraction of each oligomeric species that exists within a set of RoIs more accurately than does SpIDA. FIF achieves more accurate quantification by performing meta-analysis of brightness spectrogram distributions over different protomer-concentration ranges. The analysis consists of three stages, and it is essential that the images recorded from the solutions containing different concentrations of monomeric mEGFP are analyzed initially. We determined the molecular brightness for mEGFP molecules (205) by imaging a solution of mEGFP molecules, but focusing the laser beam on the interface between the coverslip and the solution liquid. The majority of the fluorescence signal emanates from a dense layer of mEGFP molecules attached to the surface of the coverslip by nonspecific adsorption. Because the signal emanates from a thin layer perpendicular to the axial direction of the laser beam, we used a γ factor of 0.5 (which was determined from numerical analysis) to determine the molecular brightness of the mEGFP molecules. This is the same value of γ factor found for all membrane orientations when imaging with a confocal microscope with a pinhole of 1 Airy unit (see the *γ Factor Calculation*). The mean brightness value (205 a.u.) determined from these mEGFP solution measurements was subsequently used for multiple Gaussian fitting of brightness spectrograms generated from RoIs drawn on images recorded from primary cultured neuronal cells expressing M_1_-mEGFP receptor proteins.

The key steps involved in the FIF analysis are as follows:

**Module 1** (RoI selection and segmentation): Images for analysis are combined and imported as a stack. Select the freehand polygon tool to draw multiple RoIs on each image file. The RoIs are saved and then automatically segmented to generate smaller square segments (400 pixels^2^) for spectrometric brightness and concentration analysis in module 2.
**Module 2** (quantification of RoI segment brightness and protomer concentration values): The native level of background autofluorescence intensity that is present within the imaging data needs to be measured prior to running the program. Another key parameter that needs to be input into module 2 is the monomeric brightness of the fluorophore (205), measured from RoIs drawn on fluorescence micrographs recorded from mEGFP molecules attached to the coverslip by nonspecific adsorption (Fig. 5*A*). A third important parameter is the value of the γ factor, which compensates for the nonuniformity of the laser beam’s PSF. We found that for a confocal microscope with a pinhole size of 1.0 Airy disks, the value of the γ factor was approximately 0.5, regardless of the orientation of the membrane being imaged with respect to the axial direction of the laser beam (see *γ Factor Calculation*). Other important input parameters required are reported in refs. 7 and 49. Module 2 quantifies brightness and protomer concentration values from each RoI segment by generating histogram frequency plots of the pixel fluorescent intensity values within each segment. Each segment pixel intensity distribution plot is then fitted with a single Gaussian model function and the statistical mean fluorescence intensity and SD value derived from the Gaussian fit along with the signal variance produced from the detector were used to quantify ε_eff_ and concentration values for each segment analyzed. Once all the segment-derived brightness and concentration values have been quantified, brightness frequency distributions as a function of concentration can be visualized either as a 3D surface plot of frequency of occurrence vs. concentration and ε_eff_ (which we termed “volcano graph”), or as a wire histogram plot of frequency of occurrence vs. brightness value (called a brightness spectrogram) derived from different segment bin concentration ranges.
**Module 3** (meta-analysis of brightness spectrogram distributions for various protomer concentration ranges): M_1_-mEGFP–receptor brightness spectrograms are fit with a sum of multiple Gaussian over different concentration ranges. The mean brightness values of each Gaussian peak were set as multiples of the monomeric mean brightness value measured from the mEGFP-monomer calibration solutions (205). Multiple Gaussian peak fitting of experimental M_1_-mEGFP–receptor protein brightness spectrogram data sets over a range of different protomer concentrations enabled generation of oligomer species fraction plots as a function of protomer concentration. These plots allow visualization of the different oligomeric M_1_-mEGFP–receptor protein populations in the primary neuron cultures maintained without ligand or after treatment with selected ligands.

#### γ Factor calculation.

To calculate the molecular brightness for each individual segment according to equation 1 in the Stoneman et al. report (7), we need to determine a value for the γ factor, which depends on the PSF of the illuminating beam and the geometry of the region of the sample from which signal is detected (e.g., 3D solutions vs. two-dimensional distributions of molecules such as those in membranes). In the case of a confocal microscope, γ is affected by the pinhole used in the system. The formula for the γ calculation for an imaging system using single-photon excitation can be written as follows (50):

[4] $$\gamma =\frac{∭PSF^{2}\left(x,y,z\right)dx\cdot dy\cdot dz}{∭\mathrm{PSF}\left(x,y,z\right)dx\cdot dy\cdot dz},$$

where the limits on the integrals occur over the region of the sample from which signal is detected. For example, when imaging the basolateral membrane, the fluorescent molecules are contained within approximately a10-nm layer at the plane at *z* = 0, and the value of γ is found to be 0.5, regardless of the size of the confocal pinhole placed in front of the detector. For the case when the membrane being imaged is parallel to the axial direction of the beam (i.e., a cross-sectional image of a cell), the pinhole diameter must be taken into account. For the direction parallel to the direction of the laser beam (i.e., the *z* direction) the limits on the integral in Eq. **4** can be approximated by the following equation (49–51):

[5] ωz= {[0.88⋅λEx(n−(n2−NA2)12)]2+(212⋅n⋅PHNA)2}12,

where $\lambda_{Ex}$ is the excitation wavelength, *n* is the sample’s media refractive index, NA is the objective numerical aperture, and PH is the pinhole diameter. For the plane perpendicular to the propagation of the laser beam, the limits on the integrals of Eq. **4** can be approximated as the size of the pinhole itself.

#### Quantification and statistical analysis.

Normality distributions of recovered QB values defined as MEUs were assessed by D’Agostino and Pearson normality tests (*P* > 0.05).

## Supplementary Material

Supplementary File

## Data Availability

Materials such as the M_1_-mEGFP–expressing transgenic mice have been deposited in the University of Glasgow data repository, https://doi.org/10.5525/gla.researchdata.1297. The software used for the FIF spectroscopy data analysis described in this work has been deposited in the Figshare digital repository and is accessible from https://figshare.com/s/acfd94b21b1105317f56. Video tutorials for using the software are available at the following link: https://www.youtube.com/channel/UCVjd3S28RtQ0MaxWJdNsZGA/featured. All other data are included in the manuscript and/or *SI Appendix*.
