# Supplementary material for: The M1 muscarinic receptor is present in situ as a ligand-regulated mixture of monomers and oligomeric complexes
Source: Proc Natl Acad Sci U S A. 2022 Jun 7;119(24):e2201103119. doi: 10.1073/pnas.2201103119 (PMC9214538; doi:10.1073/pnas.2201103119)
Supplement: Supplementary File [file pnas.2201103119.sapp.pdf]

PNAS
www.pnas.org

Supplementary Information for

The M_1 muscarinic receptor is present *in situ* as a ligand-regulated mixture of monomers and oligomeric complexes

Sara Marsango[1], Laura Jenkins[1], John D. Pediani[1], Sophie J. Bradley[1,3], Richard J. Ward[1], Sarah Hesse[1], Gabriel Biener[2], Michael R. Stoneman[2], Andrew B. Tobin[1], Valerica Raicu[2] and Graeme Milligan[1*]

1. **Centre for Translational Pharmacology, Institute of Molecular, Cell and Systems Biology, College of Medical, Veterinary and Life Sciences, University of Glasgow, Glasgow G12 8QQ, Scotland, U.K.**
2. **Physics Department, University of Wisconsin-Milwaukee, WI, USA**
3. **Current address: SoseiHeptares, Steinmetz Building, Granta Park, Cambridge, CB21 6DG U.K.**

Address correspondence to Graeme Milligan, Centre for Translational Pharmacology, Wolfson Link Building 253 , University of Glasgow, Glasgow G12 8QQ, Scotland, U.K. (+44 141 330 5557) (Graeme.Milligan@glasgow.ac.uk)

This file includes: Figures S1 to S4

Supporting information 1

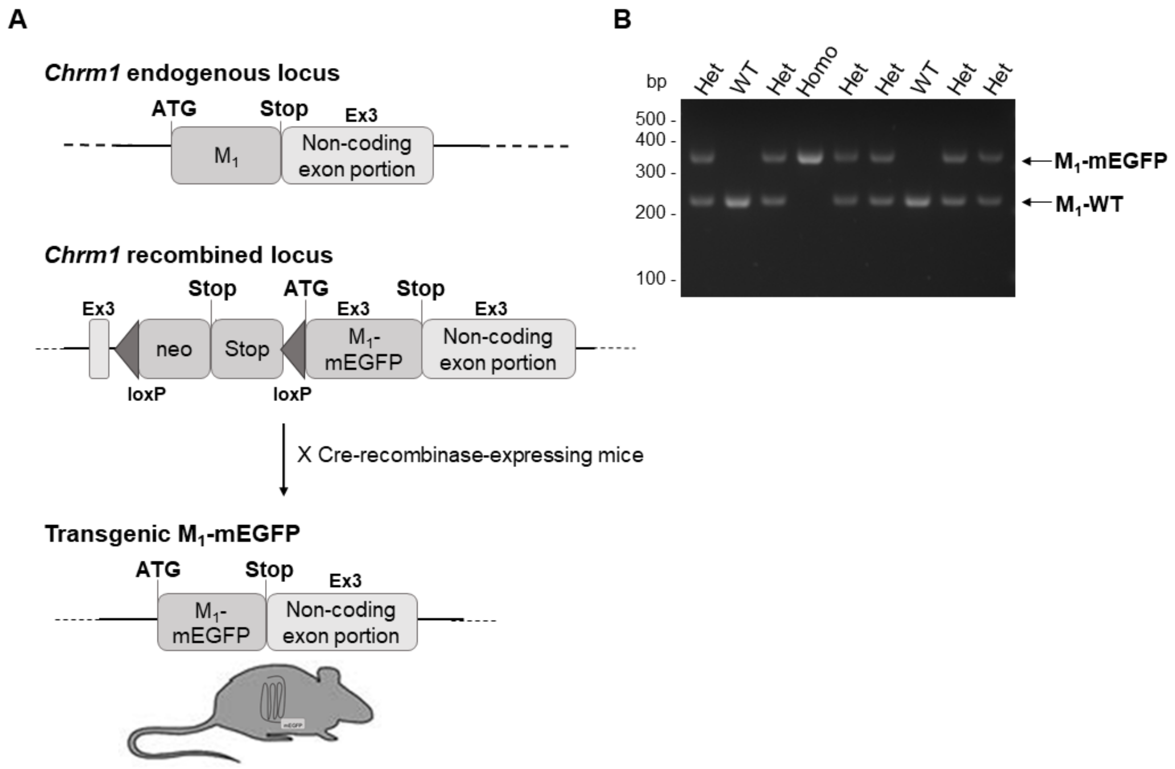

SI 1: Genotyping identifies mice both homozygous and heterozygous for expression of M_1-mEGFP

Transgenic M_1-mEGFP expressing knock-in mice were generated as illustrated (**A**). Primers (sequences in Methods) designed to amplify mouse M_1 receptor (223bp) or M_1-mEGFP (332bp) were used to PCR amplify cDNA isolated from the various mice used in these studies (**B**). WT = wild type, Het = heterozygote, Homo = homozygote M_1-mEGFP knock-in.

Supporting information 2

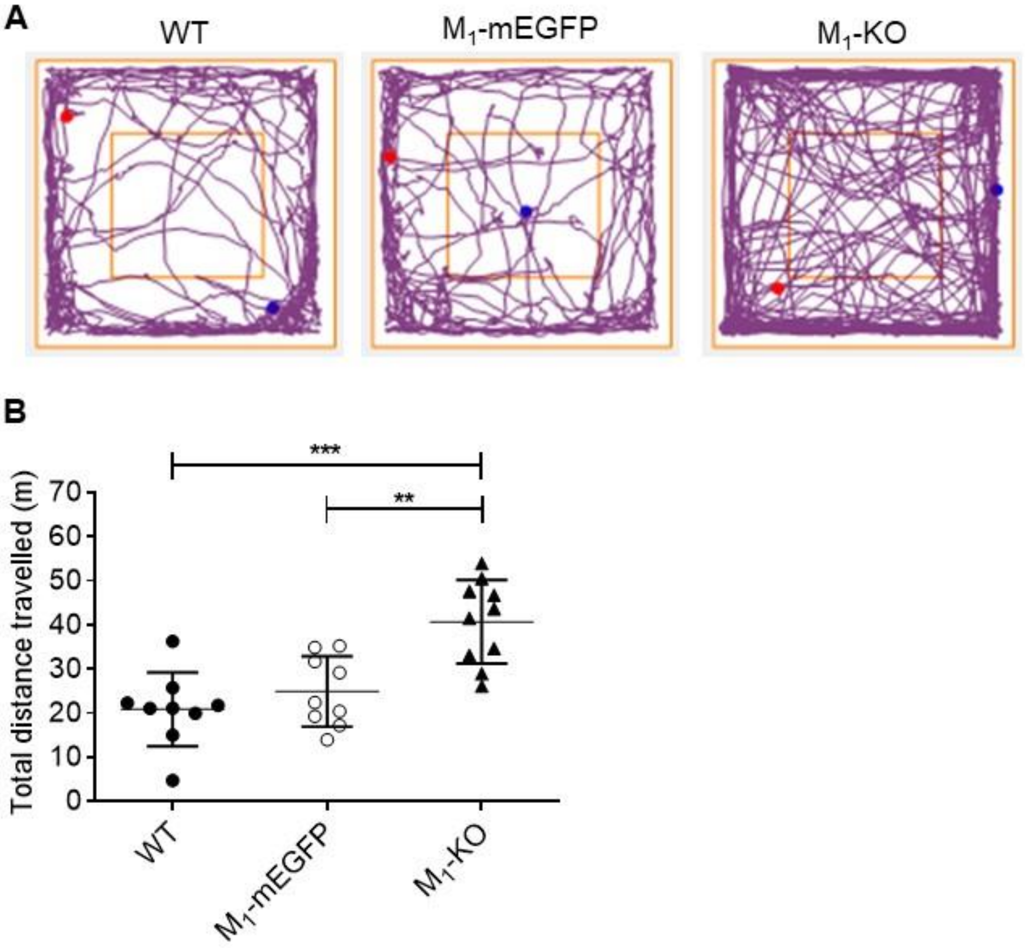

SI 2: Locomotor activity of M_1-mEGFP expressing mice is not different from wild type

Representative motion plots of wild type, homozygous M_1-mEGFP knock-in or M_1-knock-out (M_1-KO) mice in open field tests (**A**). **B**. Average distance traveled by such animals over a 10 min period in the open field test (** $P < 0.01$, *** $P < 0.001$). Data for individual animals (n = 9-10) are shown.

Supporting information 3

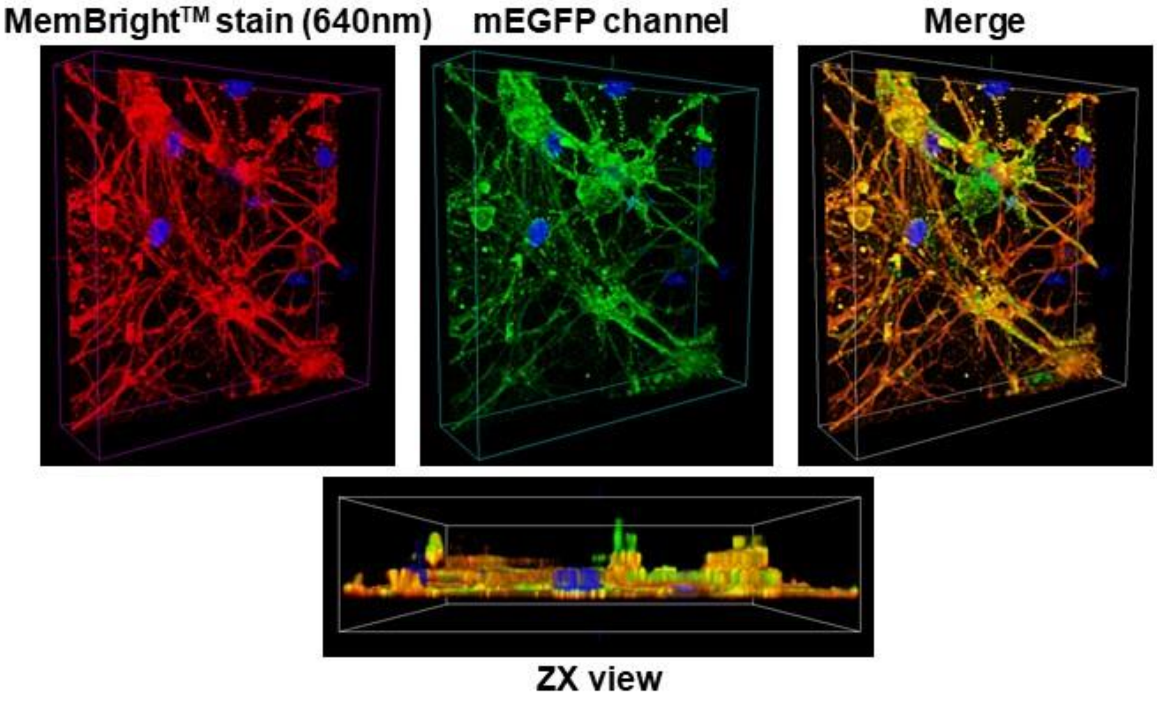

SI 3: M_1-mEGFP is present at both the cell surface and at internal locations within neurons in culture

Cultures of combined hippocampal and cortical neurons maintained for 7 days were stained with MemBrite 640™ to label cell boundaries/plasma membrane (**red**) and with Hoechst 33342 (**blue**) to identify cell nuclei. Imaging of such cultures showed that whilst some of the M_1-mEGFP (**green**) construct was present at the cell surface a significant proportion was intracellular. **A.** pseudo-3-dimensional representations. **B**. A ZX view of the same merged image as in **A.** Scale bar = 20 µm

Supporting information 4

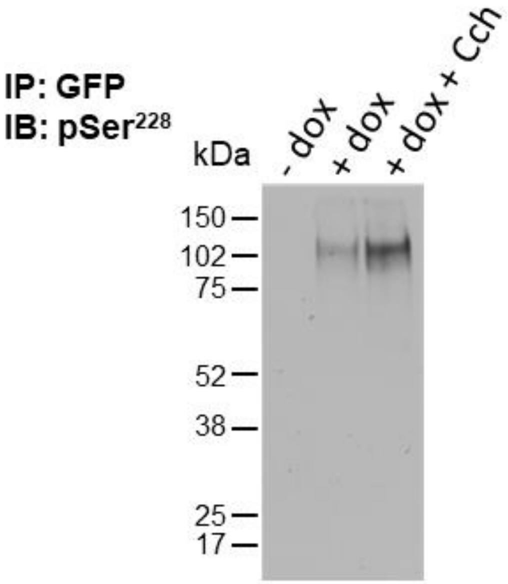

SI 4: M_1-mEGFP is phosphorylated at serine^{228} following carbachol stimulation

Flp-In TREx 293 cells harboring M_1-mEGFP (- Dox) or induced to express the receptor construct (+ Dox) were treated with 1 mM carbachol (+ Dox + Cch) for 5 min. Following enrichment of the receptor construct via GFP-trap immunoprecipitated proteins were resolved by SDS-PAGE. Immunoblot was performed with an anti-pSer^{228} M_1 antiserum (22). A representative experiment is shown.
